# Bibliometric and Visualized Analysis of Gut Microbiota and Hypertension Interaction Research Published from 2001 to 2024

**DOI:** 10.3390/microorganisms13071696

**Published:** 2025-07-18

**Authors:** Jianhui Mo, Wanghong Su, Jiale Qin, Jiayu Feng, Rong Yu, Shaoru Li, Jia Lv, Rui Dong, Yue Cheng, Bei Han

**Affiliations:** 1 School of Public Health, Health Science Center, Xi’an Jiaotong University, Xi’an 710061, China; jianhuimo@stu.xjtu.edu.cn (J.M.); suwh24@stu.xjtu.edu.cn (W.S.); qinjl@stu.xjtu.edu.cn (J.Q.); fengjiayu@stu.xjtu.edu.cn (J.F.); 3123315046@stu.xjtu.edu.cn (R.Y.); lishaoru@xjtu.edu.cn (S.L.); lvjia2017@xjtu.edu.cn (J.L.); ruidong@xjtu.edu.cn (R.D.); chengy@mail.xjtu.edu.cn (Y.C.); 2 Department of Pediatrics, The Second Affiliated Hospital of Xi’an Jiaotong University, Xi’an 710004, China

**Keywords:** hypertension, gut microbiota, research hotspots, bibliometric analysis

## Abstract

A comprehensive bibliometric analysis of literature is imperative to elucidate current research landscapes and hotspots in the interplay between gut microbiota and hypertension, identify knowledge gaps, and establish theoretical foundations for the future. We used publications retrieved from the Web of Science Core Collection (WoSCC) and SCOPUS databases (January 2001–December 2024) to analyze the annual publication trends with GraphPad Prism 9.5.1, to evaluate co-authorship, keywords clusters, and co-citation patterns with VOSviewer 1.6.20, and conducted keyword burst detection and keyword co-occurrence utilizing CiteSpace v6.4.1. We have retrieved 2485 relevant publications published over the past 24 years. A 481-fold increase in global annual publications in this field was observed. China was identified as the most productive country, while the United States demonstrated the highest research impact. For the contributor, Yang Tao (University of Toledo, USA) and the University of Florida (USA) have emerged as the most influential contributors. Among journals, the highest number of articles was published in Nutrients (n = 135), which also achieved the highest citation count (n = 5397). The emergence of novel research hotspots was indicated by high-frequency keywords, mainly “hypertensive disorders of pregnancy”, “mendelian randomization”, “gut-heart axis”, and “hepatitis B virus”. “Trimethylamine N-oxide (TMAO)” and “receptor” may represent promising new research frontiers in the gut microbiota–hypertension nexus. The current research trends are shifting from exploring the factors influencing gut microbiota and hypertension to understanding the underlying mechanisms of these factors and the potential therapeutic applications of microbial modulation for hypertension management.

## 1. Introduction

Hypertension, a chronic cardiovascular disorder characterized by persistently elevated systemic arterial pressure (≥140/90 mmHg), represents a major global health challenge [1,2]. This condition serves as a primary risk factor for stroke, ischemic heart disease, and chronic kidney disease. With hypertension and related complications claiming over 10 million lives annually, their prevention and management have emerged as critical priorities in global public health initiatives. According to the World Health Organization, it is estimated that 1.28 billion adults worldwide have hypertension. Only 46% of patients with hypertension have been diagnosed and treated, which is one of the leading causes of premature deaths worldwide [3].

Hypertension is a multifactorial disorder resulting from complex interactions between genetic and environmental factors [4]. As an interface between environmental exposures and host physiology, gut microbiota plays a pivotal role in developing various metabolic disorders, including hypertension [5]. Accumulating evidence has established a robust association between gut microbial dysbiosis and hypertension pathogenesis [4,6]. Characteristic alterations include reduced microbial diversity, increased ratio of *Firmicutes*/*Bacteroidetes*, and decreased production of short-chain fatty acids (SCFAs) such as acetate and butyrate [7,8]. Experimental studies of angiotensin II-induced hypertensive rat models have demonstrated that these dysbiotic patterns persist during hypertension development; notably, minocycline administration was shown to concurrently restore gut microbial homeostasis and attenuate blood pressure elevation [7]. These findings suggest that targeted modulation of gut microbiota may represent a promising therapeutic strategy for hypertension management through fecal microbiota transplantation, antibiotic therapy, or probiotic supplementation [9,10].

Hypertension is generally classified into two types based on its cause: primary hypertension and secondary hypertension. Secondary hypertension usually has a clear cause, such as thyroid dysfunction, chronic kidney disease, endocrine disorders, atherosclerosis, etc. [11]. This type of hypertension often shows significant improvement in symptoms after the primary cause is treated. However, in clinical practice, primary hypertension is more commonly diagnosed. The pathogenesis of primary hypertension is not fully understood yet. The mechanisms that have been confirmed by current research include abnormal renin–angiotensin–aldosterone system (RAAS), abnormal kinin–kallikrein system (KKS), vascular endothelial dysfunction, oxidative stress, inflammation, etc. [12]. Improving hypertension through the improvement of the intestinal flora can mainly be achieved by alleviating these abnormal effects. In primary hypertensive rats, it was found that the intestinal lactobacilli are significantly reduced; the CD4+, IL-17A+, and Th17 cells in the intestinal immune system are increased; and the intestinal barrier is damaged. However, when probiotics such as Bifidobacteria and short-chain fatty acid butyrate are supplemented, these conditions are improved to a certain extent [13].

Despite significant advances in understanding the interplay of gut microbiota and hypertension, the underlying molecular mechanisms and optimal therapeutic strategies remain to be fully elucidated. While many studies have explored this association, primarily focused on summarizing current findings and mechanistic hypotheses [4,14,15,16], they lack comprehensive visualization and an evolutionary analysis of research trends.

The bibliometrics constitute a quantitative analytical methodology that systematically evaluates scientific literature, including publication chronology, authorship networks, institutional affiliations, geographic distributions, and keywords evolution [17]. The rapid development of bibliometrics has led to the emergence of specialized visualization software capable of extracting and analyzing publication data to generate knowledge maps of research trends and hotspots [18] and evidence-based guidance for future research prioritization [17,19]. Here, a systematic bibliometric evaluation of gut microbiota and hypertension was applied to find valuable insights into research hotspots, knowledge gaps, and emerging directions to guide future investigations [20].

## 2. Methods

### 2.1. Data Source

The databases searched included Scopus and the Web of Science Core Collection (WoSCC) [21,22]. A review was conducted of all fully published papers that appeared in biomedical journals. It included all types of evidence, such as descriptive studies, observational studies, experimental studies, qualitative studies, and systematic reviews, in order to account for all the existing evidence. The bibliometric analysis of the literature did not include electronic publications published ahead of print, since the ultimate published dates for such publications are not known. The exported records contained complete bibliographic data in the “Plain text file” format, including the “Full Record and Cited Reference” from WoSCC, and complete bibliographic data in the “csv” format, including the full record from Scopus.

### 2.2. Search Strategy, Eligibility Criteria, and Data Refinement

**Figure 1:**
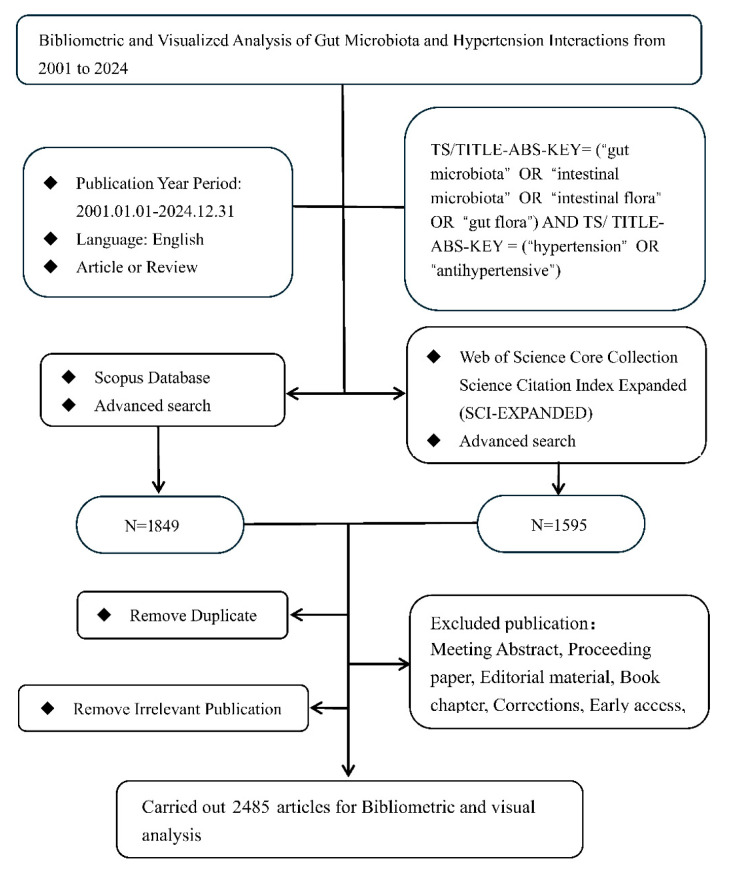
Flow chart of screening publications.

The study inclusion criteria and complete search strategy are presented in Figure 1. The literature search and selection of publications were conducted based on topic relevance, TS/TITLE-ABS-KEY = (“gut microbiota” OR “intestinal microbiota” OR “intestinal flora” OR “gut flora”) AND TS/TITLE-ABS-KEY = (“hypertension” OR “antihypertensive”). The inclusion criteria were as follows:

(1) Publication Type: “article” or “review article”;
(2) Publication Year Period: 1 January 2001–31 December 2024;
(3) Language: English;
(4) Exported Bibliographic Data: Publication title, Publication year, Author names, Country/Region, Institutional affiliations, Author Keywords, and Citation metrics.

After removing the duplicates, two investigators (JHM and WHS) independently reviewed the titles, abstracts, and references of the articles. All publication information was cross-checked, and the disagreements were resolved by a joint discussion with the senior investigators (YC and BH).

### 2.3. Data Analysis and Visualization

Bibliometric analysis employs mathematical and statistical methodologies to systematically quantify and evaluate scholarly publications. It permits comprehensive evaluation of research trajectories across multiple dimensions, including temporal trends, collaborative networks, and research hotspots [17].

GraphPad Prism 9.5.1 was utilized for analyzing annual publication trends and predicting the number of publications in 2025 using polynomials in the least squares method. VOSviewer 1.6.20 was applied for three types of network analyses (co-authorship, co-occurrence, and co-citation) [23,24] and CiteSpace 6.4.1 was employed for burst detection analysis to identify the emerging research frontiers [25]. Specifically, co-authorship analysis examined publication outputs, collaborative networks, and citation patterns among authors, institutions, and countries. The co-occurrence analysis quantified conceptual relationships through keyword frequency and cluster analysis, revealing research trends and hotspots [25,26]. Co-citation analysis traced academic networks to identify established research groups, productive regions, and influential journals, providing theoretical support for research planning and academic development [20].

During the keyword burst analysis, the emergence detection algorithm in CiteSpace 6.4.1 was able to identify burst concepts and emerging keywords at the forefront of research [27]. Betweenness centrality degree (BCD) refers to the centrality of the nodes; the higher the centrality degree, the greater the influence of the node. Nodes with BCD of more than 0.1 are usually regarded as key nodes or transformative nodes in a certain field. In VOSviewer, Total Link Strength is a comprehensive indicator that measures the strength of the connections between a certain node (such as a keyword, author, or institution) and other nodes in the network; the larger the total link strength is, the stronger the centrality of this node in the network will be [28]. In visual network diagrams, the size of the node is proportional to the number of publications or the frequency of keyword occurrences. In the publication and citation overlay map of a country/region/institution/journal, node size corresponds to publication output, with larger nodes indicating a higher number of published articles. Node color reflects the average citation frequency, where a yellow hue denotes higher citation counts. Connecting lines represent collaborations, with thicker lines signifying stronger partnerships and greater cooperative activity.

## 3. Results

### 3.1. Basic Information

**Figure 2:**
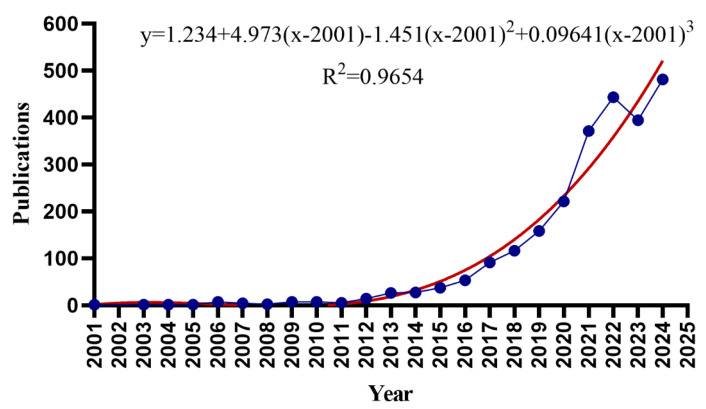
The annual publication number from 2001 to 2024.

This bibliometric analysis identified 2485 publications related to gut microbiota and hypertension, including 1803 original research articles and 682 reviews, with a total of 104,262 citations—an average of 41 citations/publication—the median citation count was 15 (with a quartile range of 5–39), only 10% of the documents had citation counts exceeding 95, while 10% of the documents had citation counts of no more than 1. The citation count range varied from 1 to 1868. The research exhibited exponential growth, with annual publications increasing from 1 in 2001 to 481 in 2024 (a 481-fold expansion). There were three distinct growth phases: pre-2011, limited activity (<10 publications/year); 2012–2021, steady growth (14 to 371 publications/year); and 2022–2024, plateau phase (439 publications/year), with a maximum of 481 publications in 2024. By 2024, over 2400 articles had been indexed in the WoSCC and Scopus, reflecting intensified scholarly interest in the research of gut microbiota and hypertension. More and more scholars are conducting related research. By 2025, the expected annual publications in this field will reach approximately 617 ± 32 (Figure 2, representing the annual publication number from 2001 to 2024).

### 3.2. Contributions of Countries/Regions to Global Publications

**Figure 3:**
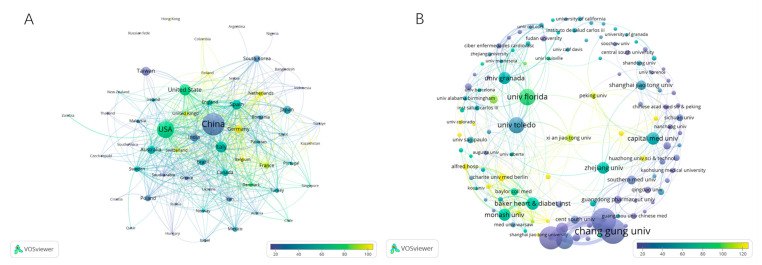
Country/region and organization analysis of the research on gut microbiota in hypertension. (**A**) Country/region co-authorship of publication and citation overlay map. Weight indicates publications, color indicates citations. (**B**) Organization co-authorship of publication and citation overlay map.

A total of 154 countries contributed publications to the research of gut microbiota and hypertension. The basic information of the top 10 countries based on the number of publications is shown in Table 1, from which it can be seen that China contributed the most articles (888, 35.73%), with most of them published in the recent years, followed by the USA (535, 21.53%), Italy (161, 6.48%) and Spain (128, 5.15%). The United States has the highest centrality (BCD = 0.57), followed by China (BCD = 0.34), Spain (BCD = 0.14), Italy (BCD = 0.12), and Germany (BCD = 0.09), suggesting that these five countries are important in the international research cooperation network (Figure 3A).

### 3.3. Author Contribution Analysis

**Figure 4:**
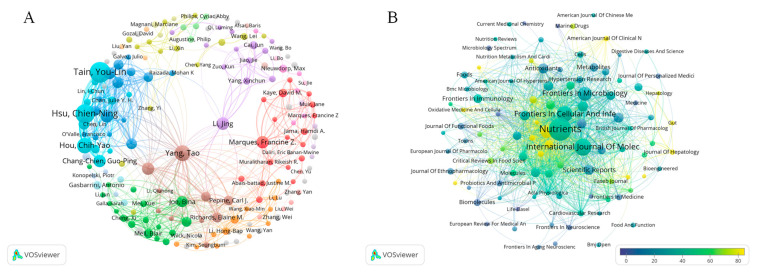
(**A**) Author co-occurrence. Circle size means the number of author-published articles. (**B**) An overlay visualization of the number of journal citations, where the size of the nodes represents citations of the journal. The color indicates the average citation, and the closer the color is to red, the higher the average number of citations for that journal.

A total of 13,765 authors contributed to these studies. The citation metrics and publication counts for the top 10 most productive authors specializing in gut microbiota and hypertension research are shown in Table 2. These leading authors collectively published 394 papers, accounting for 15.85% of all publications in this field (Figure 4A).

Citation frequency served as a key metric of author impact. In our analysis, 103 authors exceeded 1000 citations, with the highest-cited being Vijay-Kumar Matam in USA (4210 citations), Yang Tao in USA (3713 citations), Knight Rob in USA (3496 citations), Raizada, Mohan in USA (3346 citations), Pepine Carl J in USA (2723 citations), and Marques Francine in Australia (2117 citations) (Table S1). Yang Tao emerged as the most influential author, with 44 publications, and the highest citation count of 3713. Notably, only 9 out of 776,427 co-cited authors achieved citation frequencies exceeding 400. Inter-node connections represent author collaborations, where thicker lines indicate stronger collaborative relationships.

### 3.4. Institution Contribution Analysis

The literature search identified publications from 4184 organizations. Chang Gung University (Taiwan region of China) demonstrated sustained research productivity in gut microbiota and hypertension, with 72 publications from 2001 to 2024 (Figure 3B). The University of Florida (USA) emerged as the most cited institution (3858 total citations), while maintaining high productivity (43 publications, ranking fifth in output) (Figure S1). As shown in Table S2, high-output institutions were predominantly located in the Taiwan region of China, the USA, and Australia. In contrast, the most cited institutions were primarily from the USA, Germany, and Australia (Table 3). Institutions in the Taiwan region of China showed remarkable research output but a relatively lower academic impact, whereas the University of California, San Diego (USA) achieved superior research quality despite moderate output. Several institutions, including the University of Florida (USA), the University of Toledo (USA), and the Baker Heart and Diabetes Institute (Australia), excelled in both productivity and citation impact, indicating balanced performance in quantity and quality. Collaboration analysis revealed that the strongest institutional linkages are Chang Gung University, Kaohsiung Chang Gung Memorial Hospital, and Kaohsiung Medical University, as measured by total link strength. Overall, robust inter-institutional collaborations were observed across research organizations.

### 3.5. Journal Contribution Analysis

A 2458 articles were published in 831 journals in the last 24 years within the gut microbiota and hypertension field. The journal Nutrients (H-index 75) published 135 publications (5.43%), followed by the International Journal of Molecular Sciences (H-index 114, 78 publications, 3.14%) and Frontiers in Cellular and Infection Microbiology (H-index 53, 68 publications, 2.74%) (Table S3).

These journals primarily cover nutrition, biochemistry, molecular biology, and cardiovascular research. As shown in Table 4, Nutrients also received the highest total citations (5397), though with a relatively low citation average (40 citations/publication). In contrast, Hypertension achieved comparable total citations (3804) but demonstrated superior citation impact (85 citations/publication). Notably, Nature published only three articles in this field yet accumulated 3679 citations (1266 citations/publication), while *Science* received 3321 citations from two publications (1660 citations/publication), indicating exceptional research quality despite limited output in this specific domain (Figure 4B). These findings position these three journals as the most central and interconnected within this research field, suggesting their dominant scholarly influence among the 831 identified journals.

### 3.6. Analysis of Keywords

**Figure 5:**
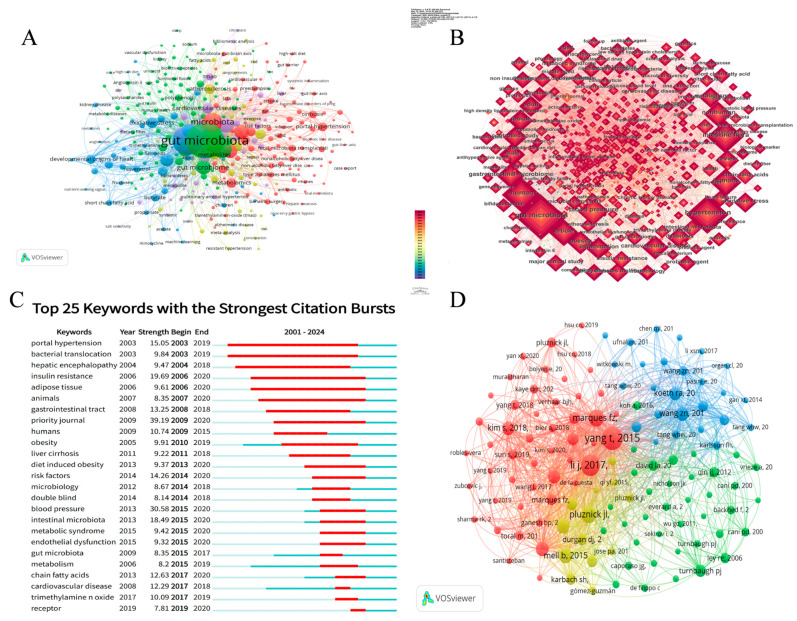
(**A**) Map of keyword clustering. (**B**) Map visualizing keyword co-occurrence. (**C**) Top 25 keywords with the highest citation bursts identified by CiteSpace. (**D**) Co-citation analysis cited reference; the size of the nodes represents the citation number of the cited reference, the same color indicates the same group, representing a high degree of correlation and similarity between the studies in these cited references.

Keywords reflect the main theme and core content of the publication; high-frequency keywords contain the research trend, implying that hot topics may appear or have appeared in this field, and provide ideas and guidance for follow-up research. As presented in Table S4, the most frequent terms were “gut microbiota” (n = 783), “hypertension” (n = 492), and “microbiota” (n = 224), which collectively define the core research themes. Secondary high-frequency keywords included “obesity” (n = 200), “inflammation” (n = 169), “probiotics” (n = 147), “blood pressure” (n = 136), “short-chain fatty acids” (n = 84), “dysbiosis” (n = 129), and “gut microbiome” (n = 122), suggesting that current research predominantly examine the gut microbiota and hypertension through inflammatory pathways, metabolic disorders, and oxidative mechanisms, while exploring the therapeutic potential of probiotics and SCFAs (Figure 5B).

The 350 high-frequency keywords were categorized into five clusters (Figure 5A). Cluster 1 (red) focused on gut microbiome research, particularly the impact of microbial dysbiosis on various diseases such as portal hypertension, metabolic syndrome, and metabolic syndrome. The dominant keywords were as follows: microbiota (n = 224), metabolic syndrome (n = 114), portal hypertension (n = 50), and insulin resistance (n = 43). Cluster 2 (green) centered on probiotics and their effects on gut health, including strain-specific functions, mechanisms of action, and synergism with prebiotics. Key terms included the following: gut microbiota (n = 783), obesity (n = 200), probiotics (n = 184), blood pressure (n = 136), and prebiotics (n = 61). Cluster 3 (blue) primarily addressed the relationship between hypertension and multiple factors, including inflammation, oxidative stress, and gut dysbiosis, along with their mechanistic roles in hypertension pathogenesis. Key terms included hypertension (n = 492), cardiovascular disease (n = 119), chronic kidney disease (n = 93), oxidative stress (n = 68), and gut dysbiosis (n = 51). Cluster 4 (yellow) examined metabolic syndrome and the related conditions (obesity, insulin resistance, diabetes, etc.) with regard to cardiovascular disease, emphasizing mechanistic pathways and potential interventions. The therapeutic potential of probiotics in modulating dysbiosis, enhancing barrier function, and immunoregulation was highlighted. Representative keywords were atherosclerosis (n = 237), diabetes (n = 237), diet (n = 218), metabolites (n = 155), nutrition (n = 123), and synbiotics (n = 48). Cluster 5 (purple) investigated SCFAs in physiological and pathological processes, particularly their roles in gut homeostasis, immunomodulation, and energy metabolism, as well as their associations with hypertension and cardiometabolic disorders. Key terms included inflammation (n = 169), dysbiosis (n = 129), short-chain fatty acids (n = 112), cardiovascular diseases (n = 67), trimethylamine N-oxide (n = 85), and bile acids (n = 25).

Essentially, keyword burst analysis aims to examine how the frequency of keyword occurrences changes over a specific time period. The results of the analysis are shown in Figure 5C. For the first five years, keywords “portal hypertension”, “bacterial translocation”, “hepatic encephalopath”, and “insulin resistance” burst out, which implies that the association between hypertension and gut microbiota likely starts with portal hypertension caused by bacterial translocation, extends to general gut flora and hypertension, and also affects complications. As the study progressed further, it began to explore factors that caused the association, with key words of “obesity”, “diet induced obesity”, “risk factors”, “double blind”, and “endothelial dysfunction”, which indicated that the investigation of the association factors gradually transitioned from the phenotype to the intrinsic mechanism and further explored the intrinsic associations on the deeper level. Studies related to gut microbiota and hypertension have focused on “trimethylamine oxide (TMAO)” and “receptors” in recent years, suggesting that the mechanisms of their association will be further extended to molecules, cells, and receptors.

### 3.7. Citation Analysis

The 2485 publications had cited 149,201 unique references, with a total citation frequency of 216,345. The first half of Table 5 presents the top 10 co-cited references, with citation counts ranging from 168 to 443. The most frequently cited reference was “Gut Dysbiosis is Linked to Hypertension” [7], with 443 citations. The 97,748 cited references were sourced from 13,057 distinct bibliographic items, including peer-reviewed journals and books. The distribution of references shows the breadth of scholarly sources in this area of research (Figure 5D).

The publication citation visualization map is shown in Figure S2, and the top 10 cited publications are shown in the second half of Table 5. Among the top ten cited publications, seven were articles and three were reviews. Of all 2485 publications, 234 were cited more than 200 times, with the most frequently cited publication being “Inflammasome-mediated dysbiosis regulates progression of NAFLD and obesity” (1868 citations), followed by “Metabolic syndrome and altered gut microbiota in mice lacking toll-Like receptor 5” and “Gastroenterology 2-inflammatory bowel disease: clinical aspects and established and evolving therapy”.

## 4. Discussion

Research on gut microbiota and hypertension has entered a phase of rapid expansion, with the annual publication count increasing from 1 article in 2001 to a cumulative total of 2485 by 2024, reflecting remarkable progress in understanding the interplay between hypertension and gut microbiota. Notably, 76% of these publications were produced within the past five years. Gut microbiota and hypertension as an interdisciplinary research field has become a captivating topic. Among the top ten most productive journals, four specialize in hypertension pathophysiology, two in nutrition, two in microbiology, one in molecular biology, and one in multidisciplinary sciences.

A total of 13,765 authors from 4184 research institutions in 154 countries/regions published 2485 articles in the WoSCC and Scopus within this field, indicating that hypertension and gut microbiota research has attracted widespread attention worldwide. While conducting independent research, countries/regions are also engaged in extensive cooperation. China demonstrated the highest research productivity with a sustained output, whereas the United States produced the highest-quality studies and occupied the central position in international collaborative networks. Consequently, both nations have emerged as leaders in advancing the research on gut microbiota and hypertension.

Cooperation among different countries is open, and there is no restriction on exchange and cooperation between institutions caused by differences in countries. Tain You-Lin and Yang Tao were identified as the most productive and most cited authors, respectively. Notably, Yang Tao demonstrated the widest research influence, with primary focus areas in cardiovascular systems, biochemistry, and molecular biology. His seminal paper “Gut Dysbiosis is Linked to Hypertension” (citation count: 1065), ranked as his most cited publication, reported that high blood pressure was linked to gut microbiota dysbiosis in both animals and humans, and that the dietary interventions to correct gut microbiota might be a novel nutritional therapy for hypertension [8].

Co-citation analysis of references enables the rapid accumulation of domain knowledge and identification of research trends in this field. The top 10 most cited references primarily focused on (1) gut dysbiosis contributing to hypertension pathogenesis, (2) microbial signaling in blood pressure regulation, and (3) alterations in gut microbiota composition and intestinal epithelial barrier integrity under hypertensive conditions. Co-occurrence analysis of keywords was employed to uncover latent knowledge structures, identify research hotspots, and delineate thematic evolution within the field [29].

Early-emerging keywords with sustained research interest included portal hypertension (2003–2019), bacterial translocation (2003–2019), hepatic encephalopathy (2004–2018), insulin resistance (2006–2020), and adipose tissue (2006–2020). Research found that intestinal bacterial overgrowth, dysbiosis, and increased mucosal permeability facilitate bacterial translocation into systemic circulation, inducing persistent inflammation [30]. Concurrently, elevated production of gut-derived toxins and bacterial metabolites (e.g., endotoxins) occurs. The liver, as the primary detoxification organ, demonstrates reduced bile secretion [31] and diminishes antibacterial capacity when chronically exposed to these toxins [32]. This metabolic impairment leads to systemic toxin accumulation, resulting in chronic low-grade inflammation, progressive hepatic fibrosis, exacerbated oxidative stress, and aggravated vascular/adipose tissue inflammation. In addition, the content of SCFAs and probiotics in the intestinal tract is greatly reduced, accompanied by a decline in the host’s immune function, which further accelerates the development of hypertension [33]. The association between hypertension and gut microbiota is likely to be observed starting with portal hypertension caused by bacterial translocation, and then this association will be extrapolated to gut flora and hypertension in general, and also that this association will affect the development of other complications.

Accumulating evidence has demonstrated that the gut microbiota plays a pivotal role in the pathogenesis of hypertension, primarily through modulation of microbial composition, intestinal barrier integrity, and microbial-derived metabolites [4]. Dysbiosis of the gut microbiota is characterized by an elevated *Firmicutes*/*Bacteroidetes* (F/B) ratio, reduced microbial diversity, depletion of beneficial bacteria, and overgrowth of pathogenic species [8]. These alterations contribute to increased enterotoxin production and systemic inflammation, ultimately compromising intestinal barrier function. Heightened intestinal permeability permits translocation of microbial pathogens and enterotoxins into systemic circulation, triggering chronic low-grade inflammation and endothelial dysfunction, leading to diminished vasodilatory factors and elevated vasoconstrictive mediators, thereby promoting the development and progression of hypertension [34]. Gut microbial metabolites, including SCFAs, TMAO, bile acids (BAs), and hydrogen sulfide (H_2_S), play critical roles in blood pressure regulation [35]. SCFAs—primarily acetate, propionate, and butyrate—modulate blood pressure through immunologic and epigenetic mechanisms by binding to G-protein-coupled receptors (GPCRs) [36]. Immunologically, SCFAs suppress the expression of pro-inflammatory cytokines, such as IL-17 and IL-6, and promote the differentiation of intestinal T cells into regulatory T cells (Tregs) via GPR43 activation, thereby attenuating excessive immune responses. Additionally, SCFAs have been shown to induce vasodilation in peripheral mesenteric arteries through the GPR41/GPR43 pathway [34]. Luo et al. demonstrated that butyrate supplementation significantly reduced blood pressure in spontaneously hypertensive rats [12]. Furthermore, butyrate has been reported to enhance β-oxidation in the intestinal mucosa [37], leading to luminal oxygen depletion and creating an anaerobic microenvironment conducive to the proliferation of beneficial gut microbiota. Trimethylamine, a choline metabolite produced by gut microbiota, is oxidized in the liver to TMAO. The main role of TMAO in the development of hypertension is not to directly cause vasoconstriction but to indirectly raise blood pressure by augmenting the prohypertensive effects of AngII [38,39]. BAs can bind to glycine or taurine to form couplers, and coupled BAs exhibit antihypertensive effects through multiple receptor pathways, e.g., BAs can activate Akt expression to increase intracellular calcium concentration and NO content, inhibit endothelin-1 (ET-1) release, and achieve vasodilatation [40]. H_2_S, as a gaseous mediator and signaling molecule, can be produced by intestinal microbiota through enzymatic reactions, and the produced H_2_S originating from the colon, after being absorbed into the intestinal blood vessels, can act as a signaling molecule to stimulate the sensory fibers of the intestinal nervous system and feedback this stimulation to the central nervous system, thus participating in the regulation of blood pressure [41]. At the same time, H_2_S also improves the endothelial dysfunction by activating the PPARδ/eNOS pathway to regulate hypertension [42]. In summary, gut microbiota is closely related to hypertension, providing new ways and directions for the prevention and management of hypertension.

Importantly, microbiota-targeted interventions, including probiotics and dietary fibers, are identified as promising therapeutic strategies for hypertension management. Experimental evidence indicates that probiotics exert beneficial effects through multiple mechanisms, including the following: (1) immunomodulation via interaction with gut-associated immune cells, stimulating enhanced antibody production and strengthening pathogen resistance; (2) microbial cross-talk with commensal microbiota, facilitating mutualistic substrate exchange, e.g., bifidobacterial cross-feeding with butyrate producers, increasing intestinal butyrate levels by 33–34% [43,44] while competitively inhibiting pathogenic colonization [45]; (3) biosynthesis of organic acids (lactate, acetate, and propionate) that acidify the intestinal lumen (pH reduction) [46], selectively inhibiting acid-sensitive pathogens (e.g., *Salmonella* spp.) while concomitantly improving cardiometabolic parameters and insulin sensitivity via SCFA-mediated pathways; (4) enhancement of intestinal barrier function through stimulated production of secretory proteins, indoles, and bacteriocins [47], with surface components (e.g., pili, flagella) acting as microbial-associated molecular patterns (MAMPs) that modulate epithelial homeostasis through pattern-recognition receptor binding [47]. These multifactorial mechanisms underlie the reported therapeutic potential of probiotics and postbiotics in diverse conditions, including dermatitis, necrotizing enterocolitis, hyperuricemia, diabetes mellitus, hypertension, and oncological pathologies [48].

In recent years, strategies to remodel the intestinal microenvironment and thereby improve hypertension through probiotics and prebiotics have gained prominence. Studies have documented the antihypertensive effects of fermented milk [49]; meanwhile, because of the important role of angiotensin I-converting enzyme (ACE) in the renin–angiotensin–aldosterone system (RAAS) and the Kinin–Bradykinin System (KKS) in the formation of hypertension [50,51,52], angiotensin I-converting enzyme (ACE) inhibitory peptides have been isolated and purified from fermented yogurt [53]. A randomized controlled trial conducted in 2003 demonstrated a significant reduction in blood pressure in hypertensive patients who consumed bioactive peptide-enriched fermented milk [54]. Although some subsequent studies have indicated that the intake of fermented yogurt is not associated with the incidence of hypertension [55], a health cohort study in 2018 pointed out that consuming at least five servings of yogurt per week could reduce the risk of hypertension by 19% [56]. However, the ability of fermented milk to reduce blood pressure requires further validation. Further research expanded the repertoire of antihypertensive peptides, with novel sources isolated from animal products, marine organisms, plants, dairy, and microbial sources [57,58,59,60,61].

## 5. Conclusions

This study presents a comprehensive bibliometric analysis of research on gut microbiota and hypertension over the past 24 years. This research has witnessed exponential growth. The current research trend in gut microbiota and hypertension is shifting from exploring the influencing factors of the development of gut microbiota and hypertension to understanding their underlying mechanisms and the potential therapeutic application of microbial modulation for hypertension management. Notably, TMAO and other receptors are identified as potential focal points for future investigations at the microbiota–hypertension interface.

## Figures and Tables

**Table 1:** Top 10 countries with the most published studies on gut microbiota and hypertension.

Rank	Country/Region	Publications (n = 2485)	Citations (n = 104,262)	Total Link Strength
1	China	888 (35.73%)	23,999	214
2	USA	535 (21.53%)	38,033	386
3	Italy	161 (6.48%)	8955	123
4	Spain	128 (5.15%)	7467	142
5	Taiwan Region of China	111 (4.47%)	2464	15
6	Australia	95 (3.82%)	4803	90
7	India	94 (3.78%)	2795	74
8	Japan	89 (3.58%)	3584	26
9	Germany	80 (3.22%)	7963	125
10	Brazil	72 (2.90%)	3114	50

**Table 2:** The top 10 authors’ publication rank in the research of gut microbiota and hypertension.

Rank	Author	Publication Count (%)	Country/Region	Institutions	Citations
1	You-Lin Tain	75 (3.02%)	Taiwan region of China	Kaohsiung Chang Gung Memorial Hospital	1852
2	Chien-Ning Hsu	66 (2.66%)	Taiwan region of China	Kaohsiung Chang Gung Memorial Hospital	1483
3	Tao Yang	44 (1.77%)	USA	University of Toledo	3713
4	Chih-Yao Hou	43 (1.73%)	Taiwan region of China	National Kaohsiung University of Science and Technology	1115
5	Jing Li	29 (1.17%)	China	Chinese Academy of Medical Sciences	1553
6	Guo-Ping Chang-chien,	28 (1.13%)	Taiwan region of China	Cheng Shiu University	562
7	Francine Marques	28 (1.13%)	Australia	Monash University	2117
8	Mohan Raizada	28 (1.13%)	USA	University of Florida	3346
9	Juan Duarte	27 (1.09%)	Spain	University of Granada	1627
10	Marta Toral	26 (1.05%)	Spain	University of Granada	1592

**Table 3:** General information about the top 10 most-cited institutions.

Rank	Institution	Documents	Citations	Total Link Strength	Country
1	University of Florida	43	3858	2719	USA
2	University of California, San Diego	11	2377	120	USA
3	Charité–Universitätsmedizin Berlin	16	2337	826	Germany
4	Monash University	30	2235	2041	Australia
5	Baker Heart and Diabetes Institute	31	2210	2003	Australia
6	Washington University	5	2181	58	USA
7	Yale University	4	2159	54	USA
8	Cleveland Clinic	9	2094	296	USA
9	German Centre for Cardiovascular Research	10	1964	734	Germany
10	Vanderbilt University	19	1947	813	USA

**Table 4:** Top 10 most-cited journals in the research field of gut microbiota and hypertension.

Journal	Citations	Publications	Citation Rank	Average Citation Rank	IF_2024_	H-Index
Nutrients	5397	135	1	9	5.0	75
Hypertension	3804	45	2	7	8.2	246
Nature	3679	3	3	3	48.5	1096
Science	3321	2	4	1	45.8	1058
International Journal of Molecular Sciences	2979	78	5	8	4.9	114
Circulation Research	2610	17	6	6	16.2	306
Frontiers in Cellular and Infection Microbiology	2447	68	7	10	4.8	53
Journal of Hepatology	2180	9	8	5	33	216
Lancet	1627	1	9	2	88.5	700
Circulation	1612	5	10	4	38.6	570

**Table 5:** The top 10 most-co-cited references and the top 10 most-cited publications.

Rank	First Author	Journal	Title	Publication Type	Citations	IF_2024_
1	Tao Yang	Hypertension	“Gut dysbiosis is linked to hypertension”	Article	443	8.2
2	Jing Li	Microbiome	“Gut microbiota dysbiosis contributes to the development of hypertension”	Article	382	12.7
3	Francine Marques	Circulation	“High-fiber diet and acetate supplementation change the gut microbiota and prevent the development of hypertension and heart failure in hypertensive mice”	Article	255	38.9
4	Pluznick Jennifer	The Proceedings of the National Academy of Sciences (PNAS)	“Olfactory receptor responding to gut microbiota-derived signals plays a role in renin secretion and blood pressure regulation”	Article	249	9.1
5	Blair R Mell	Physiological Genomics	“Evidence for a link between gut microbiota and hypertension in the Dahl rat”	Article	201	2.5
6	Monica M Santisteban	Circulation Research	“Hypertension-linked pathophysiological alterations in the gut”	Article	192	16.2
7	Adnan Sareema H	Physiological Genomics	“Alterations in the gut microbiota can elicit hypertension in rats”	Article	186	2.5
8	Zeneng Wang	Nature	“Gut flora metabolism of phosphatidylcholine promotes cardiovascular disease”	Article	178	48.5
9	Kim Seungbum	Clinical Science	“Imbalance of gut microbiome and intestinal epithelial barrier dysfunction in patients with high blood pressure”	Article	168	7.7
10	Wilck Nicola	Nature	“Salt-responsive gut commensal modulates TH17 axis and disease”	Article	168	48.5
1	Jorge Henao-Mejia	Nature	“Inflammasome-mediated dysbiosis regulates progression of nafld and obesity”	Article	1868	48.5
2	Vijay-Kumar Matam	Science	“Metabolic syndrome and altered gut microbiota in mice lacking Toll-like receptor 5”	Article	1730	45.8
3	Daniel Baumgart	Lancet	“Gastroenterology 2 -inflammatory bowel disease: clinical aspects and established and evolving therapies”	Review	1627	88.5
4	Vijay-Kumar Matam	Science	“Metabolic syndrome and altered gut microbiota in mice lacking toll-like receptor”	Article	1591	45.8
5	Clara Depommier	Nature Medicine	“Supplementation with akkermansia muciniphila in overweight and obese human volunteers: a proof-of-concept exploratory study”	Article	1428	50
6	Agustin Albillos	Journal of Hepatology	“The gut-liver axis in liver disease: pathophysiological basis for therapy”	Review	1228	33
7	Jing Li	Microbiome	“Gut microbiota dysbiosis contributes to the development of hypertension”	Article	1158	12.7
8	W.H. Wilson Tang	Circulation Research	“Gut microbiota in cardiovascular health and disease”	Review	1095	16.2
9	Tao Yang	Hypertension	“Gut dysbiosis is linked to hypertension”	Article	1083	8.2
10	Jennifer l Pluznick	The Proceedings of the National Academy of Sciences (PNAS)	“Olfactory receptor responding to gut microbiota-derived signals plays a role in renin secretion and blood pressure regulation”	Article	959	9.1

## Data Availability

The original contributions presented in this study are included in the article. Further inquiries can be directed to the corresponding author.

## Supplementary Materials

The following supporting information can be downloaded at: https://www.mdpi.com/article/10.3390/microorganisms13071696/s1, Figure S1: Organization citations map on gut microbiota and hypertension; Figure S2: Top 10 cited publications on gut microbiota and hypertension; Table S1: Top 10 authors with the most citations on gut microbiota and hypertension; Table S2: Top 10 institutions with the most publications on gut microbiota and hypertension; Table S3: Top 10 journals in terms of publications on gut microbiota and hypertension; Table S4: Top 20 Keywords of Co-Occurrence Frequency.
