# Supplementary material for: Bibliometric and Visualized Analysis of Gut Microbiota and Hypertension Interaction Research Published from 2001 to 2024
Source: Microorganisms. 2025 Jul 18;13(7):1696. doi: 10.3390/microorganisms13071696 (PMC12300951; doi:10.3390/microorganisms13071696)
Supplement: Supplementary file 1 [file microorganisms-13-01696-s001.zip › microorganisms-3740232-supplementary.pdf]

Table S1. Top 10 authors with the most citations on gut microbiota and hypertension

Table S2. Top 10 institutions with the most publications on gut microbiota and hypertension

Table S3. Top 10 journals in terms of publications on gut microbiota and hypertension

Table S4. Top 20 Keywords of Co-Occurrence Frequency

Figure S1. Organization citations map on gut microbiota and hypertension

Figure S2. Top 10 cited publications on gut microbiota and hypertension

Table S 1. Top 10 authors with the most citations on gut microbiota and hypertension

Rank	Author	Publications	Citations	Country /Region	Institutions
1	Vijay-kumar, Matam	18	4210	USA	University of Toledo
2	Tao Yang	44	3713	USA	University of Toledo
3	Rob Knight	7	3496	USA	University of California, San Diego
4	Mohan Raizada	28	3346	USA	University of Florida
5	Carl J. Pepine	17	2723	USA	National Heart, Lung, and Blood Institute
6	Francine Marques	28	2117	Australia	Baker Heart and Diabetes Institute
7	Jasenka Zubcevic,	17	1960	USA	University of Florida
8	You-lin Tain	75	1852	Taiwan region of China	Kaohsiung Chang Gung Memorial Hospital
9	Stanley Hazen	5	1851	USA	Cleveland Clinic
10	Juan Duarte	27	1627	Spain	University of Granada

Table S 2. Top 10 Institutions with the most publications on gut microbiota and hypertension

Rank	Organization	Documents	Citations	Total Link Strength	Country
1	Chang Gung University	72	1691	199	Taiwan region of China
2	Kaohsiung Chang Gung Memorial Hospital	69	1631	200	Taiwan region of China
3	Kaohsiung Medical University	65	1332	189	Taiwan region of China
4	National Kaohsiung University of Science and Technology	44	1187	149	Taiwan region of China
5	University of Florida	43	3858	52	USA
6	University of Toledo	42	1678	50	USA
7	Baker Heart and Diabetes Institute	31	2210	60	Australia
8	Capital Medical University	31	1633	34	China
9	Monash University	30	2235	61	Australia
10	Universidad de Granada	29	1674	62	Spain

Table S 3. Top 10 journals in terms of publications on gut microbiota and hypertension

Rank	Journal	Documents	Citations	Total Link Strength	IF_{2024}	H-index
1	Nutrients	135	5397	868	4.8	75
2	International Journal of Molecular Sciences	78	2979	528	4.9	114
3	Frontiers in Cellular and Infection Microbiology	68	2447	312	4.6	53
4	Frontiers in Microbiology	52	1405	213	4	88
5	Hypertension	45	3804	1150	7.2	246
6	Scientific Reports	28	493	110	3.8	149
7	Antioxidants	27	451	235	6	---
8	Frontiers in Nutrition	27	479	122	4	---
9	Frontiers in Physiology	27	1022	260	3.2	75
10	Frontiers in Endocrinology	26	755	36	3.9	51

Table S 4. Top 20 Keywords of Co-Occurrence Frequency

Rank	Keyword	Occurrences	Cluster	Rank	Keyword	Occurrences	Cluster
1	gut microbiota	783	6	11	microbiome	117	1
2	hypertension	492	2	12	metabolic syndrome	114	1
3	microbiota	224	1	13	chronic kidney disease	93	2
4	obesity	200	3	14	short-chain fatty acids	84	5
5	inflammation	169	5	15	oxidative stress	68	2
6	probiotics	147	3	16	cardiovascular diseases	67	5
7	blood pressure	136	6	17	atherosclerosis	61	4
8	dysbiosis	129	5	18	prebiotics	61	3
9	gut microbiome	122	6	19	diabetes	59	4
10	cardiovascular disease	119	2	20	diet	59	4

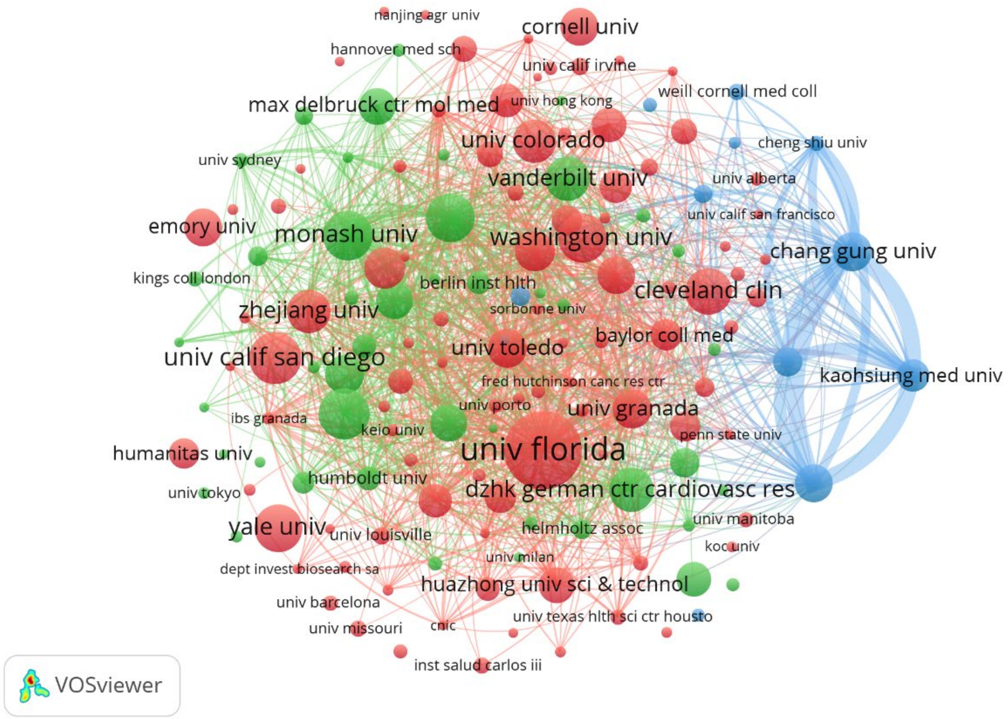

Figure S 1. Organization citations map on gut microbiota and hypertension

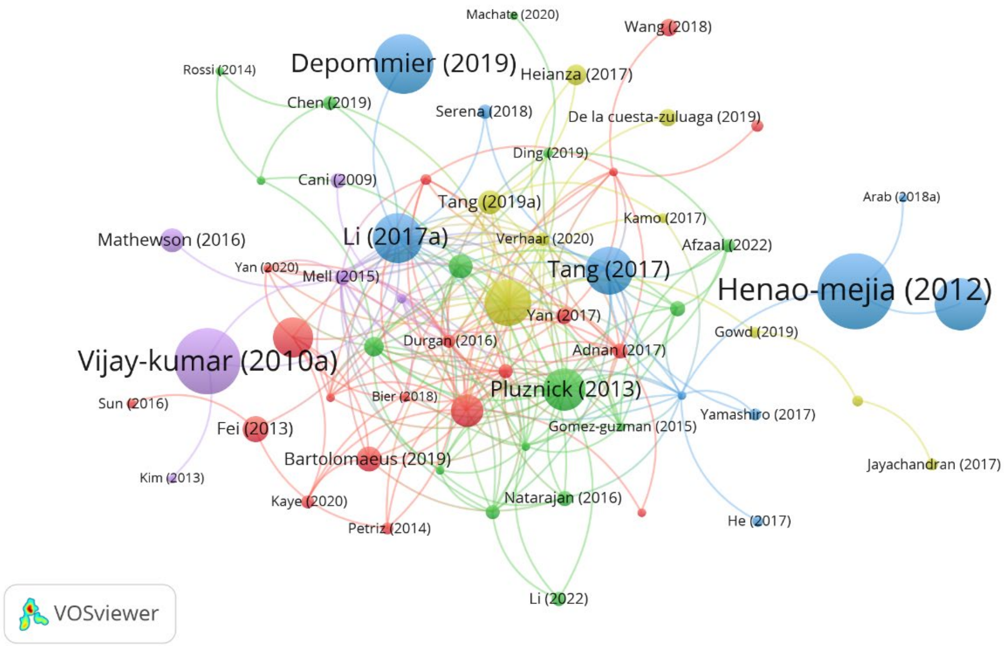

Figure S2. Top 10 cited publications on gut microbiota and hypertension
